# Ensemble Learning with Multiclassifiers on Pediatric Hand Radiograph Segmentation for Bone Age Assessment

**DOI:** 10.1155/2020/8866700

**Published:** 2020-10-27

**Authors:** Rui Liu, Yuanyuan Jia, Xiangqian He, Zhe Li, Jinhua Cai, Hao Li, Xiao Yang

**Affiliations:** ^1^Department of Medical Informatics, Chongqing Medical University, Chongqing 401331, China; ^2^Chengdu Second People's Hospital, Chengdu 610017, China; ^3^Department of Radiology, Children's Hospital Affiliated to Chongqing Medical University, Chongqing 400014, China; ^4^Department of Mechanical and Electrical Engineering, University of Electronic Science and Technology, Chengdu 611731, China

## Abstract

In the study of pediatric automatic bone age assessment (BAA) in clinical practice, the extraction of the object area in hand radiographs is an important part, which directly affects the prediction accuracy of the BAA. But no perfect segmentation solution has been found yet. This work is to develop an automatic hand radiograph segmentation method with high precision and efficiency. We considered the hand segmentation task as a classification problem. The optimal segmentation threshold for each image was regarded as the prediction target. We utilized the normalized histogram, mean value, and variance of each image as input features to train the classification model, based on ensemble learning with multiple classifiers. 600 left-hand radiographs with the bone age ranging from 1 to 18 years old were included in the dataset. Compared with traditional segmentation methods and the state-of-the-art U-Net network, the proposed method performed better with a higher precision and less computational load, achieving an average PSNR of 52.43 dB, SSIM of 0.97, DSC of 0.97, and JSI of 0.91, which is more suitable in clinical application. Furthermore, the experimental results also verified that hand radiograph segmentation could bring an average improvement for BAA performance of at least 13%.

## 1. Introduction

Automatic bone age assessment (BAA) based on the hand radiographs is a crucial diagnostic technique to evaluate the growth disorders and endocrine abnormalities for pediatric and adolescent patients, usually performed by radiological examination of the left hand and the wrist radiographs to assess skeletal maturity in clinical [1–3]. The Greulich and Pyle (G&P) method [4] and the Tanner-Whitehouse (TW3) method [5] are two most widely used traditional methods for bone age estimation. But both of them are time-consuming and subjective. Therefore, the automatic evaluation of bone age based on computing power and machine learning techniques, especially the application of deep Convolutional Neural Networks (CNNs), has been studied and prompted the development of the BAA [6].

**Figure 1:**
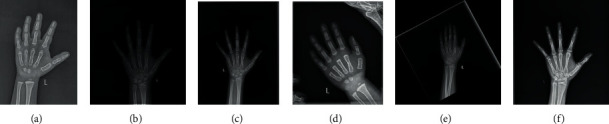
Different types of hand radiographs in the whole datasets, including (a) overexposure, (b) low contrast, (c) uneven radiation intensity, (d) irrelevant bones, (e) frame attach to the bone marked by the radiologist, and (f) radioactive markers.

In the processing pipeline of automated BAA, image preprocessing, segmentation, and normalization were shown to be effective for improving the robustness and performance of BAA models in the previous studies [7–10], and the most important of which is hand bone segmentation [11], which could seriously affect the prediction accuracy. The hand bone segmentation could remove all extraneous objects, such as radioactive markers, impurities, and noise, and extract the whole hand. Medical image segmentation is a necessary but a challenging problem in most image analysis and classification problems. In the process of digital radiograph acquisition, an intrinsic effect will be caused when radiation intensities exposed unevenly on the examined subject [12, 13]. Owing to the influence of the uneven radiation intensity and various man-made factors, most hand radiographs have motion artifacts, noise, and asymmetric illumination. The representative examples are shown in Figure 1 that most images have low contrast and blurring edge, which is complicated to extract the entire hand bone region from the background.

In the previous studies, the most widely utilized traditional segmentation techniques could be divided into the following several kinds according to the different image characteristics, thresholding, clustering, edge based, region based, deformable models, and hybrid techniques [14–16], but these methods frequently result in oversegmentation, especially the distal phalanx. When dealing with large datasets, the robustness limitations of traditional segmentation methods are even more pronounced. Therefore, the deep learning techniques were introduced to medical image segmentation [17], and patch-based CNN pixel classification is one of which the most popular segmentation methods [18]. LeNet-5 network was the first published application of using patch-based CNN to segment the hand and wrist [19]. In this study, 1000 radiographs were classified into sample patches to train the detection network. But because of patches' overlap, the network was really time-consuming. The U-Net network [20], which was originally proposed for medical image segmentation and could be utilized for segmentation problems with limited amounts of data [21], was applied to predict hand masks [22]. Another network VGG-16 [23] was integrated with U-Net as an encoder-decoder structure to obtain hand mask [24]. However, U-Net network required multiple trainings for binary image segmentation, and most predicted label maps had false-positive regions assigned to the hand class. Manual labor was needed to clean these masks and trained the model again for six times. Deep CNNs have been gradually devoted in medical image segmentation, but they showed weak efficiency for automatic hand radiograph segmentation in recent researches. Moreover, it was a great amount of work to creating labels of the training datasets for CNN. As a result, it is necessary to design a segmentation method with low complexity and strong processing capability.

Aiming at the problems mentioned above, we proposed utilizing a model to predict the optimal segmentation threshold for hand mask segmentation, which was trained on multiclassifiers based on ensemble learning. We also compared the proposed method with the representatively used traditional segmentation techniques and the U-Net network.

## 2. Materials and Methods

**Figure 2:**
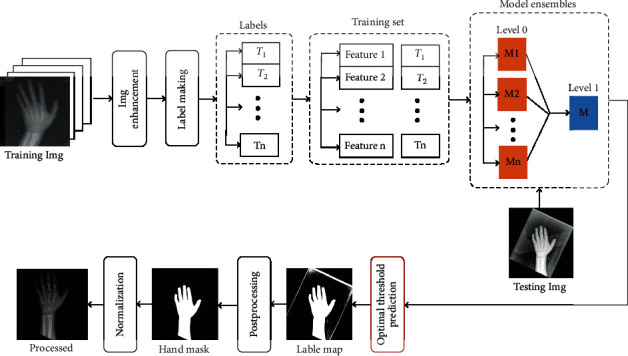
Procedure of engine to automatically segment hand bone image.

In this section, we described our approach for hand radiograph segmentation, and the whole procedure was illustrated as Figure 2. The main idea of the proposed method consisted of four stages: (1) image enhancement using the histogram equalization, (2) label making of optimal segmentation threshold, (3) a 2-level ensemble learning of classification model training based on multiple classifiers, and (4) postprocessing through region growing for clean hand masks.

### 2.1. Image Enhancement

Image enhancement is very essential to improve the segmentation performance and robustness of the image processing [25]. The histogram equalization method is an efficient way to enhance the contrast and smooth the histogram for hand radiographs [26, 27]. Generally, the histogram with obvious double peaks is well suited to the selection of image optimal threshold, while the rest of the histograms are the opposite with several small peaks needed to be processed to restore contrast, and the optimal thresh value could be easily found to create hand mask in this way.

### 2.2. Label Making

The main steps to find the optimal segmentation threshold can be summarized as below:

*Step 1*: Chose 40 values at the interval of 2 below average to obtain binary image. The selection range of threshold value could be increased to a limited extent if no correct value was available.

**Figure 3:**
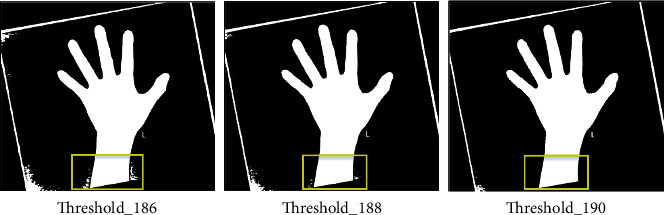
The selection of the optimal segmentation threshold.

*Step 2*: Selected the threshold value with the best segmentation result as the training label. The optimal threshold should meet the following two conditions: first, the background is completely separated from the palm, and moreover, the details of hand masks are exquisite. Try to choose the threshold with a larger value as the label, and as shown in Figure 3, threshold value of 190 was marked as the label. Especially, the selected optimal segmentation thresholds were corrected by three people with professional background without interference.

*Step 3*: Calculated the histogram, mean, and variance gray value of each image as the feature and utilized the features and labels as the training dataset.

*Step 4*: To eliminate the adverse effect caused by the outliers. The training set was standardized by min-max normalization.

(1) $$\begin{array}{c} \left[\begin{array}{c} {f_{i}}^{\prime }\\ {l_{i}}^{\prime } \end{array}\right]=\frac{\left[\begin{array}{c} f_{i}\\ l_{i} \end{array}\right]-\min\left(I_{i}\right)}{\max\left(I_{i}\right)-\min\left(I_{i}\right)}, \end{array}$$

where *I*_*i*_ is the pixel value, *f*_*i*_ is the feature, and *l*_*i*_ is the label of each image. By this way, the deviation is finally normalized to (0, 1).

**Figure 4:**
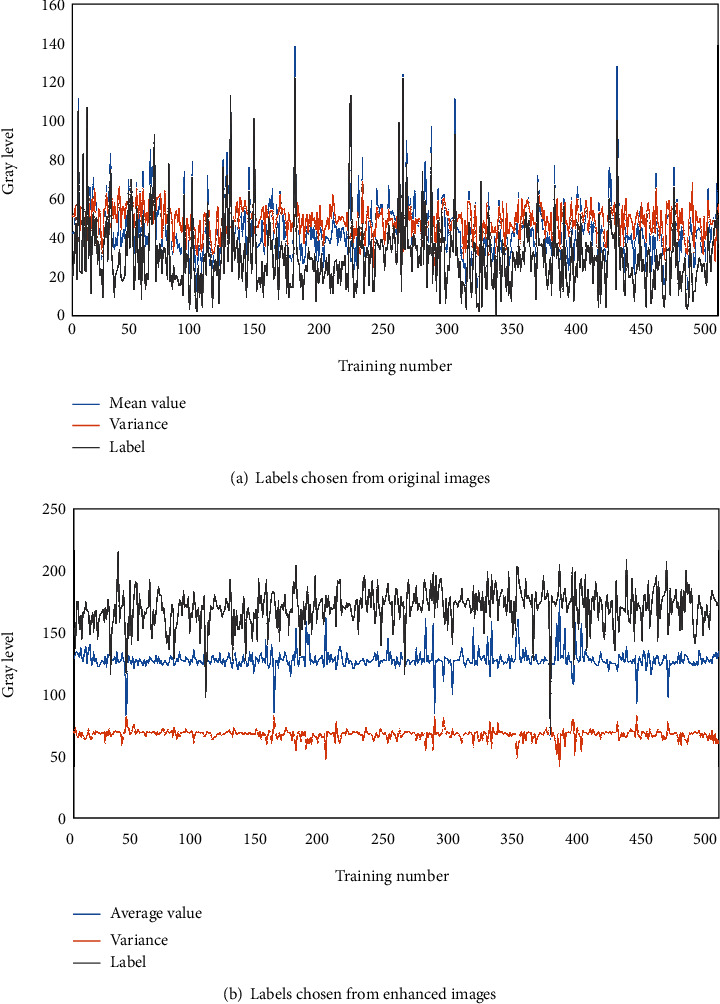
Label distribution chosen artificially from the training set: (a) labels chosen from original images and (b) labels chosen from enhanced images.

Figure 4 shows the distribution of sample labels chosen artificially from the training set. As can be seen, almost all the optimal threshold values were limited in 150 and 200 after enhanced processing. This was to suggest that the enhanced processing made the distribution of the labels more uniform, which was able to be less susceptible to the impact of imbalanced samples on model fitting.

### 2.3. Ensemble Learning Framework

**Figure 5:**
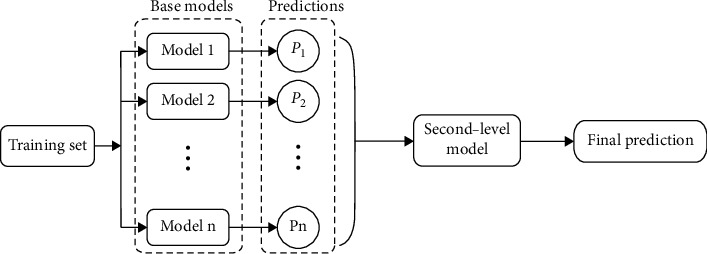
The main idea of the stacking technology.

Individual classifier may not be able to learn more information, while ensemble learning can improve the performance of a single classifier by combining them [28]. Ensemble learning is one of the most useful strategies to improve generalization performance of prediction model, with a core of training strategy for base classifiers, such as bagging, boosting, and stacking [29]. Bagging and boosting build the base learners from a single dataset, having an impact on diversity, while stacking learning method uses the multiple classifiers by taking the prediction of the previous level as input variables for the next level [30]. Therefore, stacking learning strategy is considered to construct the ensemble learning framework for hand segmentation. The simplified flow diagram of stacking algorithm was shown in Figure 5.

To get a good ensemble, the base learners should be accurate and diverse. It is generally recognized that the diversity among base classifiers is important for improving generalization performance in an ensemble of model. We examined the ability of various classifiers, aiming to choose the most effective one as the base learner. The performance of each model was evaluated by determining the root mean square error (RMSE) between the predictions and labels, and the grid search method was employed to optimize parameters; the optimization details were shown as follows:

*SVC*(class_weight = ‘balanced', degree =2, gamma =0.1, kernel = ‘sigmoid', max_iter = -1, random_state =5),

*DecisionTreeClassifier*(max_depth =3, max_features = ‘auto', splitter = ‘best'),

*RandomForestClassifier*(max_features=‘auto', n_estimators=150, oob_score=True, max_depth=200),

*ExtraTreesClassifier*(class_weight = ‘balanced', bootstrap = True, max_features = ‘sqrt', random_state =30, n_estimators =100, oob_score = True),

*GaussianNB*(),

*XGBClassifier*(gamma=0, learning_rate=0.01, max_depth=3, n_estimators=200, objective=‘multi:softmax'),

*KNeighbors*Classifier(n_neighbors =5, weights = ‘distance'),

*BaggingClassifier*(max_features=0.5, n_estimators=100, oob_score=True, random_state=50),

*GradientBoostingClassifier*(learning_rate = 0.01, max_features = ‘sqrt', n_estimators =200, subsample = 0.6),

*AdaBoostClassifier*(learning_rate =0.1, n_estimators =50, random_state =30).

The performance of each base classifier was shown in Table 1.

(2) $$\begin{array}{c} \text{RMSE}=\sqrt{\frac{1}{m}\overset{m}{\underset{i=1}{\sum }}{\left(y_{i}-{\overset{\wedge }{y}}_{i}\right)}^{2}.} \end{array}$$

**Figure 6:**
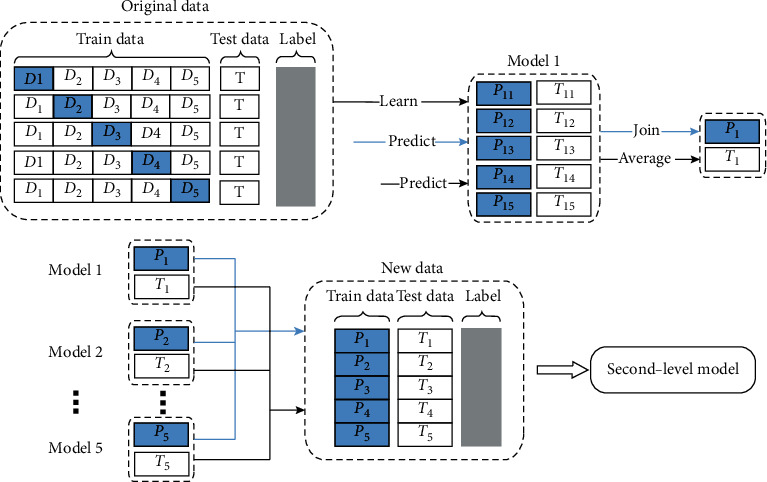
Ensemble learning using stacking technology based on 5-fold cross validation.

Considering the computing cost, we decided to select the top five classifiers, RandomForest, ExtraTrees, Bagging, AdaBoost, and SVC as the base learner of the stacked model. To increase the diversity of base classifiers, we applied 5-fold cross validation in the training process, which was illustrated in Figure 6.

*Step 1*: The training set was randomly divided into *D*_1_, *D*_2_, ⋯*D*_5_subsets with similar size. Defined $\overset{-}{D_{i}}=D/D_{i}$ as the training set and *D*_*i*_ as the testing set when base model training, where *i* = {1, 2, 3, 4, 5} and *D* = {*D*_1_, *D*_2_, *D*_3_, *D*_4_, *D*_5_}. The whole testing set was denoted as *T*.

*Step 2*: Trained the model 1 by $\overset{-}{D_{1}}$and made a prediction *P*_11_ by*D*_1_. Such operations were needed to be repeated five times, and thus we could get a new training set of $P_{1}=\left(\begin{array}{c} P_{11}\\ \vdots \\ P_{15} \end{array}\right)$.

*Step 3*: The whole testing set *T*was predicted by the base model 1 trained on $\overset{-}{D_{i}}$ in every 5-fold cross validation and made a prediction *T*_11_, *T*_12_, *T*_13_, *T*_14_, *T*_15_, respectively. Thus, a new testing set *T*_1_could be obtained in computing five predictions, $T_{1}=\left(\begin{array}{c} T_{11}\\ \vdots \\ T_{15} \end{array}\right)$.

For the base model 2 to model 5, repeated steps 2 to steps 3 until the training set *P*_2_, *P*_3_, *P*_4_, *P*_5_ and testing set *T*_2_, *T*_3_, *T*_4_, *T*_5_were achieved. Predictions provided by each base model were combined into a new training set *P* = (*P*_1_, ⋯, *P*_5_) and a new testing set *T*′ = (*T*_1_, ⋯, *T*_5_). Imported *P*and *T*′to the second-level model and the labels remained the same as original dataset.

The choice of second-level model is equally important, and compared with other classifiers, Logistic regression is the most often choice. For best performance, we also considered Softmax regression and the best performing base classifier RandomForest shown in Table 1 to make a comparison, and the results were shown in Table 2. From the chart, we knew that the Softmax regression performed better with a RMSE of 6.47 than the Logistic regression and the RandomForest. Therefore, Softmax regression was chosen as the second-level model for the ensemble learning of stacking.

### 2.4. Postprocessing

**Figure 7:**
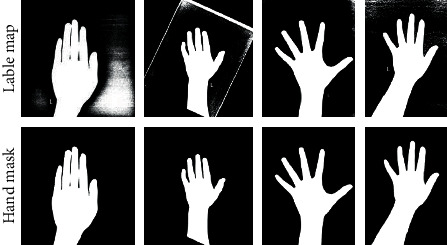
The postprocessing of the hand mask.

There could appear to be false-positive pixels in the hand label maps predicted by a stacker model, so we extracted the hand area through region growing. The center of the image was taken as the seed, and the growth was stopped in the edge of the hand shape. As a result, a clean mask could be created for the hand radiograph. The postprocessing of the hand mask was shown in Figure 7.

### 2.5. Evaluation Metrics

The objective evaluation of the proposed method mainly depends on a series of quantitative parameters. Peak signal to noise ratio (PSNR) [31], structural similarity (SSIM) [32], dice similarity coefficient (DSC), and Jaccard similarity index (JSI) [33] were commonly used to calculate the errors between the segmented images and the ground truth. PSNR and SSIM are both image quality evaluation indexes, while the DSC and JSI are segmentation accuracy assessment indexes.

PSNR can be computed using the equation as

(3) $$\begin{array}{c} \text{PSNR}=10*\log_{10}\left(\frac{255^{2}}{\text{MSE}}\right). \end{array}$$

MSE is denoted as

(4) $$\begin{array}{c} \text{MSE}=\frac{1}{256\times 256}\overset{256}{\underset{i=1}{\sum }}\overset{256}{\underset{j=1}{\sum }}{\left(S-G\right)}^{2}, \end{array}$$

where *S* stands for segmented image and *G* for ground truth of the segmented image.

SSIM measures the similarity of two images, is defined as

(5) $$\begin{array}{c} \text{SSIM}=\frac{\left(2\mu_{S}\mu_{G}+C_{1}\right)\left(2\sigma_{SG}+C_{2}\right)}{\left({\mu_{S}}^{2}+{\mu_{G}}^{2}+C_{1}\right)\left({\sigma_{S}}^{2}+{\sigma_{G}}^{2}+C_{2}\right)}, \end{array}$$

whenre *μ*_*S*_ and *σ*_*S*_^2^ are the mean and the variance of the segmented image, respectively. Likewise, *μ*_*G*_and *σ*_*G*_^2^ are the mean and the variance of the ground truth mask, respectively. And *σ*_*SG*_ is the covariance of the predicted mask and the ground truth mask. *C*_1_ and *C*_2_ are both constants to retain the stability of numerator and denominator.

DSC is defined as

(6) $$\begin{array}{c} \text{DSC}=2\frac{\left|S\cap G\right|}{\left|S+G\right|}. \end{array}$$

JSI is given by equation

(7) $$\begin{array}{c} \text{JSI}=\frac{\left|S\cap G\right|}{\left|S\cup G\right|}. \end{array}$$

Except the index PSNR, the range of value for other metrics is 0 to 1, where 1 demonstrates the perfect segmentation result.

## 3. Experiments and Results

A set of experiments implemented on hand radiograph segmentation were designed to verify the effectiveness of the proposed method. To ensure the fairness of the experiments, the histogram equalization method was carried on the training and testing datasets in all comparative methods. All the experiments were performed on a CPU environment, python3.6, and Tensorflow 1.11.0.

### 3.1. Datasets

In this study, a total of 600 hand radiographs with the skeletal age ranging from 1 to 18 years old were included into the whole dataset. We randomly selected 500 images as the training set and 100 images as the testing set. These whole 600 hand masks ground truth images were manually labeled by professional radiologists. The dataset was all collected and anonymized from the radiology department of Children's Hospital Affiliated to Chongqing Medical University.

### 3.2. Strategy Testing

**Figure 8:**
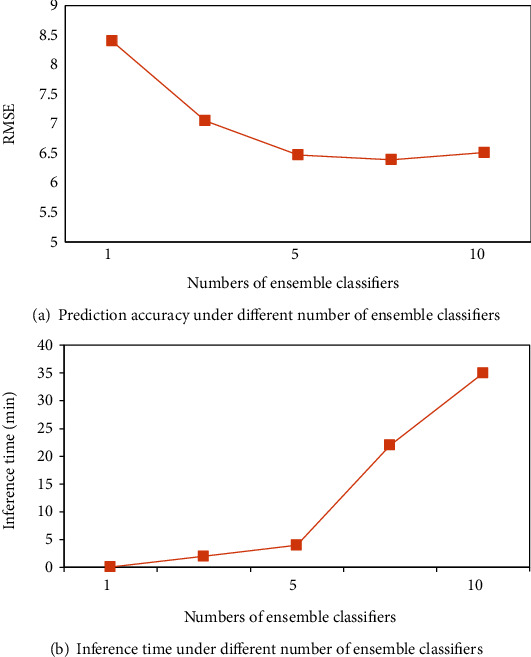
Sensitivity to the number of ensemble classifiers: (a) prediction accuracy under different number of ensemble classifiers and (b) inference time under different number of ensemble classifiers.

To verify the effectiveness of 5 independent ensemble classifiers with stacking, it is necessary to evaluate whether the number of multilevel models affects the accuracy of the stacked model. Therefore, we designed several experiments to measure prediction accuracy as well as inference time under different ensemble classifiers for comparison. Each time, we selected the best performing classifiers as the base learners for stacking when changing the ensemble number, such as when the number was set as 2, the top two classifiers, RandomForest and ExtraTrees were used as the combination. When the ensemble number was set as 3, RandomForest, ExtraTrees, and Bagging classifiers were selected as base learners and so on. The results are detailed in Figure 8; we used RMSE to measure the prediction accuracy.

As shown in Figure 8, the ability of model fitting accuracy and inference time were greatly affected under different component classifiers in an ensemble. With the increasing number of ensemble classifiers, the prediction accuracy was significantly improved, but increased more slowly when the number was greater than five, even became worse when the number was approximate ten. Moreover, the inference time became longer as the number of component classifiers increased, especially when the number exceeded 5, and 35-minute inference time for 5 ensemble classifiers was reasonable compared to other configurations. Therefore, an ensemble of 5 classifiers for optimal segmentation threshold prediction based on stacking proposed in this research was proved to be effective, either the model performance or computational complexity.

### 3.3. Qualitative Analysis

To verify the effectiveness of the proposed approach, we made a comparison about the performance between the proposed method and three representative traditional segmentation approaches Otsu thresholding [34], K-means clustering [35], and GrabCut [36] from previous researches, as well as the U-Net network, which is the most common method for hand bone segmentation in deep learning. We used an open source tool in deep learning named Labelme to make the ground truth images of hand radiographs, and each image took approximately 3 minutes to delineate. U-Net was trained by binary_crossentropy loss function with Adam optimizer. We used 500 images for training the network with 20 epochs. The learning rate was set as 1e-4, and each step used a batch size of 2 images.

**Figure 9:**
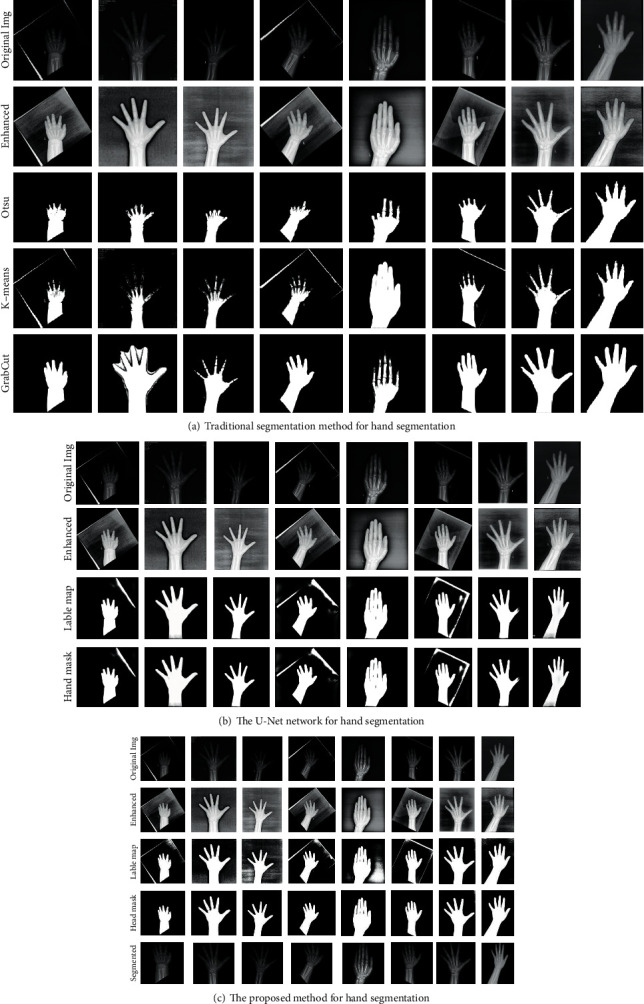
Hand segmentation performance based on different methods: (a) traditional segmentation method for hand segmentation, (b) the U-Net network for hand segmentation, and (c) the proposed method for hand segmentation.

The segmentation results were shown in Figure 9. As we can see from Figure 9(a), the classical traditional segmentation algorithm, Otsu, K-means, and GrabCut had resulted in undersegmentation of the phalanges, especially the Otsu thresholding and K-means clustering. The hand masks predicted by the U-Net network, as shown in Figure 9(b), were a little worse than our method, because some clean hand masks could not be extracted by the label map predicted by the network.  Figure 9(c) demonstrated the effectiveness of the proposed entire segmentation engine. The hand masks could be separated by extracting the connected region through region growing from black backgrounds by the predicted optimal threshold. We also cropped and resized the segmented image appropriately to 512 × 512, as shown in the last line. As a result, we were able to get a final segmented hand radiographs using the generated clean hand mask.

### 3.4. Quantitative Analysis

As shown in Table 3, the proposed method outperformed other three representative traditional methods as well as the U-Net network on segmentation accuracy, achieving a DSC of 0.97 and JSI of 0.93. And the SSIM, which was for image quality measurement, also showed the best result with an average value of 0.97. Although the PSNR of our method with an average value of 54.37 dB was slightly worse than the U-Net with the maximizing value of 55.92 dB, it is significantly better than the traditional methods, Otsu, K-means, and GrabCut with an average value of 42.54, 41.62, and 46.87, respectively. Even more important, in reference to time complexity, we could see that U-Net network had offered the longest runtime of 3400 minutes of any other tests performing on a CPU environment. Otsu algorithm showed the superiority in time complexity of 8 minutes, while other index values were least unsatisfactory. Consequently, our method with 20-minute computing time was comparatively acceptable.

### 3.5. Impact of Hand Segmentation for BAA

**Figure 10:**
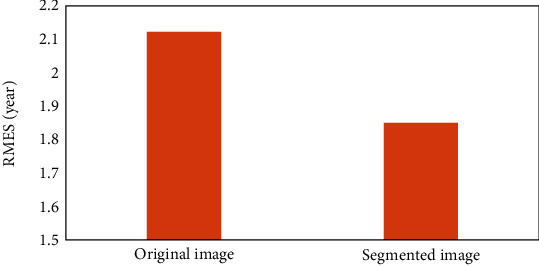
Performance of bone age assessment under different configurations.

To demonstrate that the proposed hand segmentation method can improve the accuracy of BAA, we chose the VGG16 as our training model to make a comparison. This network was one of the most common used models in the research of BAA. We marked the bone ages of dataset of 500 total images in years; hence, there were 19 classes overall. Due to the small dataset, the pretrained weights from Imagenet were used to initialize the weights and then the vgg16 was fine tuned with these weights. We also used data augmentation including rotation, translation, scaling, and shifting by keras 2.2.4. Softmax cross entropy was applied as the loss function to optimize the model with Adam optimizer. The training data contained 90% of the original dataset, while the validation set contained the rest. The learning rate was set as 0.01, and each step used a batch size of 2 images. The bone age assessment results under different configurations are shown in Figure 10. As can be seen from the diagram, compared with the BAA constructed by the original image, there was a performance increase of average 13% in RMSE of the BAA based on the segmented image; RMSE decreased from 2.12 years to 1.85 years, which suggested that the proposed hand segmentation method could effectively improve the accuracy of the BAA. It is believed that the accuracy improvement for BAA brought by the hand segmentation will become more apparent with more hand radiograph images.

## 4. Discussion

We have proposed an effective method which has good adaptability and generalization for hand radiograph segmentation in this paper. We find that (1) the proposed approach outperforms commonly used traditional methods and a state-of-the-art architecture U-Net on a small dataset, (2) and hand segmentation can effectively improve the forecast precision of bone age assessment. To this end, various experiments were carried out to validate the effectiveness and practicability of our method.

From the strategy testing, as shown in Figure 8, greater emphasis had been placed on the number of component classifiers for better executive speed and the generalization capacity using ensemble learning. The predictive ability of single model is not as strong as that of ensembles. When the number of component classifier was set as 5, RMSE was 6.47, and when we increased the component number to 7, the RMSE decreased from 6.47 to 6.39. While when the ensemble number was set as 10, the RMSE increased to 6.51. Therefore, there was a slow increasing of model performance when the ensemble number was set more than 5, but a decrease of RMSE when the number was set more than 7. Generalization error and over fitting problem might be caused by the excessive model ensemble. Moreover, the inference time was nearly 9 times when the number was set as 10 compared with 5 classifiers. Therefore, both the segmentation accuracy and execution speed should be taken into consideration in an ensemble learning.

Figure 9 and Table 3 show the segmentation results between the proposed method and other methods. The proposed method performs better in hand bone segmentation with robust and highest segmentation accuracy. With regard to the traditional methods, though simple, the segmentation accuracy was unsatisfactory. Compared with the U-Net network, our method took the advantage of the segmentation accuracy and the time complexity. The U-Net took 200 times computing time than ours on a CPU. As for the impact of hand segmentation for BAA, it was obvious that there was a better performance in RMSE with the overall hand-segmented images, which suggested that the hand image segmentation step was important for generalizability of the BAA model.

In a word, the traditional segmentation methods with weak robust and low precision have not been applicable for hand mask segmentation. Although the U-Net has the unstable performance in recognition of the edge of the hand and a great deal of training time, it is still the most popular technology in dealing with many segmentation tasks. However, deep learning lies in the massive and complicated task to artificially annotate the ground truth images for model training, and repeated training process is required to get better prediction results from a small dataset. More importantly, deep neural networks require powerful operation ability of the computer, like a GPU. By contrast, our method can be trained in a small dataset and taken in a considerable computational cost in CPU. No matter what the quality or the accuracy of the segmented image, our method has obtained the satisfactory results, which is superior to the traditional segmentation methods and the U-Net network in deep learning obviously.

The study in this paper still has some limitations even if the good segmentation results have been obtained. The input features for multiple classifiers, normalized histogram, mean value, and variance of each image can be made several optimizations to improve the model fitting ability and meanwhile, boost efficiency. In addition, our experiments are only conducted on hand radiographs, and different types of images can be used to test the generalization ability of this method. Otherwise, there are some special hand radiographs with variable collimation configurations digitized from traditional film Digital Radiography (DR) could not be satisfied segmented based on our method or deep learning. Therefore, it will be the main topic of the research in future work.

## 5. Conclusions

In this work, we have proposed an automatic hand radiograph segmentation method based on ensemble learning with multiclassifiers, which can effectively improve the overall performance for BAA. We converted the process of searching for optimal threshold into a classification task. Ensemble learning with 5-fold stacking strategy was utilized to train the classification model. Demonstrated by the experimental results and analysis, the proposed method greatly contributed to improvements on the performance of optimal segmentation threshold prediction, resulting in better accuracy for hand mask segmentation using a small dataset, which was more effective in clinical application.

## Figures and Tables

**Table 1:** The performance of each base classifier.

Classifiers	RMSE
SVC	9.89
DecisionTree	11.14
RandomForest	8.40
ExtraTrees	8.63
GaussianNB	10.80
XGB	10.87
KNeighbors	18.67
Bagging	8.88
GradientBoosting	15.50
AdaBoost	9.37

**Table 2:** Performance of stacked model based on different second-level classifiers.

Second-level classifier	RMSE
Logistic regression	8.82
Softmax regression	6.47
RandomForestClassifier	7.69

**Table 3:** Quantitative evaluation comparison of our proposed method and other methods on 100 testing set.

Methods		PSNR (dB)	SSIM	DSC	JSI	Time (min)
Traditional methods	Otsu	42.54	0.88	0.72	0.47	8
	K-means	41.62	0.86	0.70	0.52	250
	GrabCut	46.87	0.88	0.85	0.71	188
U-Net		55.92	0.95	0.88	0.68	3400
Our method		54.37	0.97	0.97	0.93	20

## Data Availability

All hand radiographs used in this work are available from the corresponding author on request.

## References

[1] Spampinato C., Palazzo S., Giordano D., Aldinucci M., Leonardi R. (2017). Deep learning for automated skeletal bone age assessment in X-ray images. *Medical Image Analysis*.

[2] Tajmir S. H., Lee H., Shailam R. (2019). Artificial intelligence-assisted interpretation of bone age radiographs improves accuracy and decreases variability. *Skeletal Radiology*.

[3] Booz C., Wichmann J. L., Boettger S. (2019). Evaluation of a computer-aided diagnosis system for automated bone age assessment in comparison to the Greulich-Pyle atlas method. *Journal of Computer Assisted Tomography*.

[4] Garn S. M. (1959). Radiographic atlas of skeletal development of the hand and wrist. *American Journal of Human Genetics*.

[5] Morris L. L. (2003). Assessment of skeletal maturity and prediction of adult height (TW3 method). *Journal of Medical Imaging and Radiation Oncology*.

[6] Larson D. B., Chen M. C., Lungren M. P., Halabi S. S., Stence N. V., Langlotz C. P. (2018). Performance of a deep-learning neural network model in assessing skeletal maturity on pediatric hand radiographs. *Radiology*.

[7] Ren X., Li T., Yang X. (2018). Regression convolutional neural network for automated pediatric bone age assessment from hand radiograph. *IEEE Journal of Biomedical and Health Informatics*.

[8] Iglovikov V., Rakhlin A., Kalinin A., Shvets A. Pediatric bone age assessment using deep convolutional neural networks. http://arxiv.org/abs/1712.05053.

[9] Halabi S. S., Prevedello L. M., Kalpathy-Cramer J. (2019). The RSNA pediatric bone age machine learning challenge. *Radiology*.

[10] Siegel E. L. (2019). What can we learn from the RSNA pediatric bone age machine learning challenge?. *Radiology*.

[11] Milletari F., Navab N., Ahmadi S. A. V-net: fully convolutional neural networks for volumetric medical image segmentation.

[12] Stolojescu-Crisan C., Holban S. (2013). A comparison of X-ray image segmentation techniques. *Advances in Electrical and Computer Engineering*.

[13] Jacob N. E., Wyawahare M. V. (2013). Tibia bone segmentation in X-ray images - a comparative analysis. *International Journal of Computer Applications*.

[14] Su L., Fu X., Zhang X. (2018). Delineation of carpal bones from hand X-ray images through prior model, and integration of region-based and boundary-based segmentations. *IEEE Access*.

[15] Waseem Khan M. (2014). A survey: image segmentation techniques. *International Journal of Future Computer and Communication*.

[16] Simu S., Lal S. (2017). A study about evolutionary and non-evolutionary segmentation techniques on hand radiographs for bone age assessment. *Biomedical Signal Processing and Control*.

[17] Kumar E. S., Bindu C. S. Medical image analysis using deep learning: a systematic literature review.

[18] Hesamian M. H., Jia W., He X., Kennedy P. (2019). Deep learning techniques for medical image segmentation: achievements and challenges. *Journal of Digital Imaging*.

[19] Lee H., Tajmir S., Lee J. (2017). Fully automated deep learning system for bone age assessment. *Journal of Digital Imaging*.

[20] Ronneberger O., Fischer P., Brox T. U-Net: convolutional networks for biomedical image segmentation.

[21] Iglovikov V., Mushinskiy S., Osin V. Satellite imagery feature detection using deep convolutional neural network: a kaggle competition. http://arxiv.org/abs/1706.06169.

[22] Pan X., Zhao Y., Chen H., Wei D., Zhao C., Wei Z. (2020). Fully automated bone age assessment on large-scale hand X-ray dataset. *International Journal of Biomedical Imaging*.

[23] Simonyan K., Zisserman A. Very deep convolutional networks for large-scale image recognition. http://arxiv.org/abs/1409.1556.

[24] Chu M., Liu B., Zhou F., Bai X., Guo B. Bone age assessment based on two-stage deep neural networks.

[25] Chou K. Y., Lin C. S., Chien C. H., Chiang J. S., Hsia C. H. Using statistical parametric contour and threshold segmentation technology applied in X-ray bone images.

[26] Yao L., Muhammad S. (2019). A novel technique for analysing histogram equalized medical images using superpixels. *Computer Assisted Surgery*.

[27] Wu E., Kong B., Wang X., Jun B., Yi L., Feng G. Residual attention based network for hand bone age assessment.

[28] Guo Z., Li X., Huang H., Guo N., Li Q. (2019). Deep learning-based image segmentation on multimodal medical imaging. *IEEE Transactions on Radiation and Plasma Medical Sciences*.

[29] Agarwal S., Chowdary C. R. (2020). A-stacking and A-bagging: adaptive versions of ensemble learning algorithms for spoof fingerprint detection. *Expert Systems with Applications*.

[30] Low C. Y., Park J., Teoh A. B. (2019). Stacking-based deep neural network: deep analytic network for pattern classification. *IEEE Transactions on Cybernetics*.

[31] Hore A., Ziou D. Image quality metrics: PSNR vs. SSIM.

[32] Wang Z., Bovik A. C., Sheikh H. R., Simoncelli E. P. (2004). Image quality assessment: from error visibility to structural similarity. *IEEE Transactions on Image Processing*.

[33] Chang H., Zhuang A. H., Valentino D. J., Chu W. (2009). Performance measure characterization for evaluating neuroimage segmentation algorithms. *NeuroImage*.

[34] Ostu N. A. (1979). A threshold selection method from gray-level histograms. *IEEE Transactions on Systems, Man, and Cybernetics*.

[35] Shan P. (2018). Image segmentation method based on K-mean algorithm. *EURASIP Journal on Image and Video Processing*.

[36] Han S., Tao W., Wang D., Tai X. C., Wu X. (2009). Image segmentation based on GrabCut framework integrating multiscale nonlinear structure tensor. *IEEE Transactions on Image Processing*.

